# Quantitative characterization of androgen receptor protein expression and cellular localization in circulating tumor cells from patients with metastatic castration-resistant prostate cancer

**DOI:** 10.1186/s12967-014-0313-z

**Published:** 2014-11-26

**Authors:** Edwin E Reyes, David J VanderWeele, Masis Isikbay, Ryan Duggan, Alexa Campanile, Walter M Stadler, Donald J Vander Griend, Russell Z Szmulewitz

**Affiliations:** Department of Surgery, University of Chicago, Chicago, IL USA; Committee on Immunology, University of Chicago, Chicago, IL USA; Department of Medicine, University of Chicago, Chicago, IL USA; Flow Cytometry Facility, University of Chicago, Chicago, IL USA

**Keywords:** Prostate neoplasms, Androgen receptor, Circulating tumor cells, Castration-resistant prostate cancer

## Abstract

**Background:**

Many current therapies for metastatic castration-resistant prostate cancer (mCRPC) are aimed at AR signaling; however, resistance to these therapies is inevitable. To personalize CRPC therapy in an individual with clinical progression despite maximal AR signaling blockade, it is important to characterize the status of AR activity within their cancer. Biopsies of bone metastases are invasive and frequently fail to yield sufficient tissue for further study. Evaluation of circulating tumor cells (CTCs) offers an alternative, minimally invasive mechanism to characterize and study late-stage disease. The goal of this study was to evaluate the utility of CTC interrogation with respect to the AR as a potential novel therapeutic biomarker in patients with mCRPC.

**Methods:**

Fifteen mL of whole blood was collected from patients with progressive, metastatic mCRPC, the mononuclear cell portion was isolated, and fluorescence-activated cell sorting (FACS) was used to isolate and evaluate CTCs. A novel protocol was optimized to use ImageStreamX to quantitatively analyze AR expression and subcellular localization within CTCs. Co-expression of AR and the proliferation marker Ki67 was also determined using ImageStreamX.

**Results:**

We found inter-patient and intra-patient heterogeneity in expression and localization of AR. Increased AR expression and nuclear localization are associated with elevated co-expression of Ki-67, consistent with the continued role for AR in castration-resistant disease. Despite intra-patient heterogeneity, CTCs from patients with prior exposure to abiraterone had increased AR expression compared to CTCs from patients who were abiraterone-naïve.

**Conclusions:**

As our toolbox for targeting AR function expands, our ability to evaluate AR expression and function within tumor samples from patients with late-stage disease will likely be a critical component of the personalized management of advanced prostate cancer. AR expression and nuclear localization varies within patients and between patients; however it remains associated with markers of proliferation. This supports a molecularly diverse AR-centric pathobiology imparting castration-resistance.

**Electronic supplementary material:**

The online version of this article (doi:10.1186/s12967-014-0313-z) contains supplementary material, which is available to authorized users.

## Background

Metastatic prostate cancer is the 2nd-leading cause of cancer mortality among American men [1] and is associated with significant morbidity in addition to loss of life. Androgen deprivation and other methods to disrupt androgen receptor (AR) signaling were the first targeted cancer therapy and have been the mainstay of prostate cancer therapy for more than seven decades [2]. Two recently approved therapies for advanced prostate cancer directly target the androgen receptor signaling pathway: abiraterone by preventing androgen synthesis, and enzalutamide by blocking interaction of the receptor with its ligands. There is even evidence that docetaxel, the first approved systemic therapy with a demonstrated survival benefit, acts by disrupting AR function [3].

Though most men with prostate cancer initially respond to these AR-targeted therapies, resistance is inevitable. As with other targeted therapies, alteration or modification of the target is a likely resistance mechanism and a number of such mechanisms have already been described including upregulation of AR expression, as well as mutation and deletion of the AR ligand binding domain [4-9]. Nevertheless, translational studies, including assessment of clinical relevance of various AR alterations, have been hampered by a lack of access to tissue. Prostate cancer typically metastasizes to bone, and biopsy of these lesions is invasive, painful, and low yield [10]. A few programs have instituted warm autopsy programs, collecting samples of metastatic disease shortly after willing patients die from their disease [11]. These programs are rare, however, and offer only a final snapshot of the biology of resistant disease.

Circulating tumor cells (CTCs) are emerging as a source of therapy-resistant prostate cancer material, a so-called “liquid biopsy” that could serve as a therapeutic biomarker [12,13]. CTCs can be obtained through relatively non-invasive means, opening the door to serial assessments of the disease state and examine tumor cell response to therapy. In 2008 the FDA approved the use of the CELLSEARCH system (Janssen Diagnostics) to collect and enumerate CTCs in patients with prostate cancer [14]. With this system, there is a clear relationship between the number of CTC’s and disease prognosis. Additional methods for CTC isolation have been developed, including microfluidic techniques [15], RT-PCR detection [16], fluorescence in situ hybridization (FISH) [17], and enrichment based approaches using other magnetic devices [18-21], lipid content [22], size [23,24], or charge. Many of these technologies are confounded by white blood cell (WBC) contamination, are often dependent on fixed cells, have focused on CTC enumeration, and have not necessarily been conducive to molecular interrogation of isolated cells.

We sought to develop and utilize techniques that allow flexibility in translational application. The eventual goal of our research is to provide support for CTC protein interrogation as a predictive therapeutic biomarker for the personalization of mCRPC care. As AR is the pivotal molecular driver of prostate cancer evolution and progression and is the foremost therapeutic target for mCRPC, our current study focused on AR interrogation. To that end we used fluorescence activated cell sorting (FACS)-based methods allowing for protein analysis and collection of live cells. We focused our approach using ImageStreamX, which combines microscopy techniques with flow cytometry. ImageStreamX captures each cellular event in a digital photograph and calculates multiple parameters, including fluorescence intensity and location, within each captured image [25-27]. This flexibility and specificity allowed an integrated evaluation of AR within CTCs in patients with metastatic castration-resistant prostate cancer (mCRPC).

## Methods

### Patients

All patients were treated at the University of Chicago for mCRPC and provided informed consent per an Institutional Review Board-approved prospective clinical protocol. All patients were progressing on their current therapy by PSA or radiologic criteria. Blood (15 mL) was collected and processed within 2 hours, including isolation of mononuclear cell fraction (buffy coat). Two cohorts were evaluated: our initial cohort of FACS-sorted CTCs evaluated by immunofluorescent microscopy, and a second cohort of 20 patients with CTCs evaluated by ImageStreamX.

### Isolation of CTCs by FACS

Patient blood was drawn into blood collection tubes (BD: 362753). Isolated mononuclear cells were stained with either an Alexa-488 conjugated EpCAM antibody (Biolegend, 1:100) or a PE-conjugated EpCAM antibody (Biolegend, 1:100) and a QDot800 conjugated CD45 antibody (Invitrogen, 1:100). CTCs were isolated using a MoFlo XDP flow-sorting machine to sort for EpCAM positive/CD45 negative cells.

### Protein expression by immunofluoresence (IF)

Isolated CTCs that were utilized for protein expression analysis via immunofluoresence (IF) were sorted into 8-chambered slides (LAB-TEK: 154941). CTC cells were stained with combinations of the following primary antibodies overnight and secondary antibodies: Androgen Receptor (Santa Cruz, N-20 antibody, 1:100) or an Alexa-647 conjugated AR antibody (Cell Signaling, AR-XP, 1:100), PSA (Santa Cruz 7638 1:50), PE conjugated pan-cytokeratin (Santa Cruz 8018, 1:50), DyLight 680 anti-rabbit secondary (KPL, 1:100), Dylight 594 anti-goat (KPL, 1:100). DAPI containing mounting media was used to coverslip slides (Vector laboratories: H1200). Slides were visualized with the Leica SP2 laser scanning confocal microscope and images were analyzed using ImageJ software.

### Targeted DNA sequencing

To extract and amplify DNA, sorted cells were collected into PCR tubes containing 3 μl PBS and DNA extracted using REPLI-g (Qiagen) following the manufacturer’s instructions for amplification from single cells. To sequence a portion of the AR gene, primers were used to amplify exons 7 and 8, and the forward primer used for capillary sequencing. (AR intron7 F GAGGCCACCTCCTTGTCAACCCTG and AR 3UTR R2 GGCACTGCAGAGGAGTAGTGCAGA).

### ImageStreamX analysis

#### Analysis of CTCs from patients with mCRPC

Buffy coat obtained from patients with mCRPC for ImageStreamX analysis was centrifuged at 450 G for 10 minutes and washed with PBS. Cells were blocked using FcR Blocking Reagent (Miltenyi Biotec, 1:10) for 10 minutes. One μl of Biotin anti-human CD45 (Biolegend, 1:100) per 5 × 10^6^ cells was added, incubated for 20 minutes, washed with PBS, and centrifuged for 10 minutes at 450 G. This was repeated twice. Anti-biotin microbeads (Miltenyi, 1:4) were added to cells at a 1:4 dilution per 10^7^ cells for 15 min, washed with PBS, and centrifuged at 450 G. Cells were re-suspended in 500 μl PBS and CD45 depleted using AutoMACS Pro (Miltenyi) per the manufacturer’s instructions. Following depletion, cells were centrifuged for 10 minutes at 450 G, washed and fixed for 15 minutes with 3.2% Ultra Pure EM Grade Formaldehyde (Polysciences, Inc., 1:6), and stored at 4C for up to three months (average storage time was 4 weeks). Staining was performed using EpCAM (Biolegend;1:40), and CD45 (Life Technologies; 1:27) antibodies in the dark for 30 minutes. For intracellular staining, cells were then washed with PBS. Fix/perm buffer from FoxP3 Buffer Set (eBioscience, 1:4) was added to cells for 30 minutes then washed off with PBS. Next, cells were washed with Perm Buffer from FoxP3 Buffer Set (eBioscience, 1:10) and centrifuged at 450 G for 5 minutes. Cells were stained intracellularly with AR (Cell Signaling, 1:11), and Ki-67 (Biolegend, 1:11) in the dark for one hour. Cells were washed with Perm Buffer and then PBS. Finally, cells were stained with FxCycle Violet (Invitrogen, 1:1000) and acquired on ImageStreamX (Amnis). Single stain control with gaiting strategy for each antibody is shown in Additional file 1: Figure S1. Gating strategies and multi-marker compensation were maintained. Analysis was conducted using IDEAS software (Amnis).

#### Comparison of staining fixed versus unfixed cells

One million LAPC-4 cells were fixed for 15 minutes with 3.2% Ultra Pure EM Grade Formaldehyde (Polysciences, Inc.). Fixed cells and an equal number of unfixed LAPC-4 cells were washed with 1× PBS and centrifuged for 5 minutes at 450 G. Following centrifugation, cells were stained with EpCAM (Biolegend, 1:40) for 30 minutes at 4C then washed with PBS. Both fixed and unfixed cells were analyzed via flow cytometry. FACSDiVa Software was used for data acquisition and FlowJo software was used for analysis.

#### Determination of Nuclear versus Cytoplasmic AR localization

LAPC-4 cells were cultured in serum starved media. On day 1, either 1 μM R1881 or 10 μM of enzalutamide was added to the cells. On day 2 cells were intracellularly stained for AR as described above.

#### Single stain compensation controls

VCAP or CWR22RV1 cells were fixed for 15 minutes with 3.2% Ultra Pure EM Grade Formaldehyde (Polysciences, Inc.). Cells were washed with PBS, centrifuged for 5 minutes, and stained for EpCAM (Biolegend, 1:40), CD45 (Life Technologies, 1:27), AR (Cell Signaling, XP conjugated antibody, 1:11), Ki-67 (Biolegend, 1:11) and FxCycle Violet (Invitrogen, 1:1000). Cells were acquired using ImageStreamX (Amnis; Seattle, WA) and analyzed using IDEAS software (Amnis; Seattle, WA).

#### Evaluation of cell proliferation

A mitomycin dose response curve was performed on CWR22RV1 between 1 ug/ml to 8 ug/ml, and cells were incubated for 4 hours. The media was replaced, and the cells were incubated overnight. Cells were stained for Ki-67 and analyzed by ImageStreamX.

### Statistical analyses

For our initial feasibility cohort, a detection rate of 35% was postulated as the minimum threshold of feasibility given the reported detection rate for CTCs between 50 and 70%. Thus, a minimum of six of the first fifteen analyzed patients needed to have isolated and visualized CTCs to satisfy our feasibility threshold and allow the study to continue.

A student’s *t* test was used to analyze the difference in Ki-67 expression following exposure to Mitomycin. A Wilcoxon signed-rank test was used to analyze the association between Ki-67 and AR expression, and between Ki-67 and similarity index. A Wilcoxon rank sum test was used to analyze the association between AR expression and prior exposure to abiraterone, and between similarity index and prior exposure to abiraterone. A mixed model with a random patient effect was used to analyze the difference in area between EpCAM+ and EpCAM- cells.

## Results

### Feasibility of CTC isolation and interrogation

**Figure 1:**
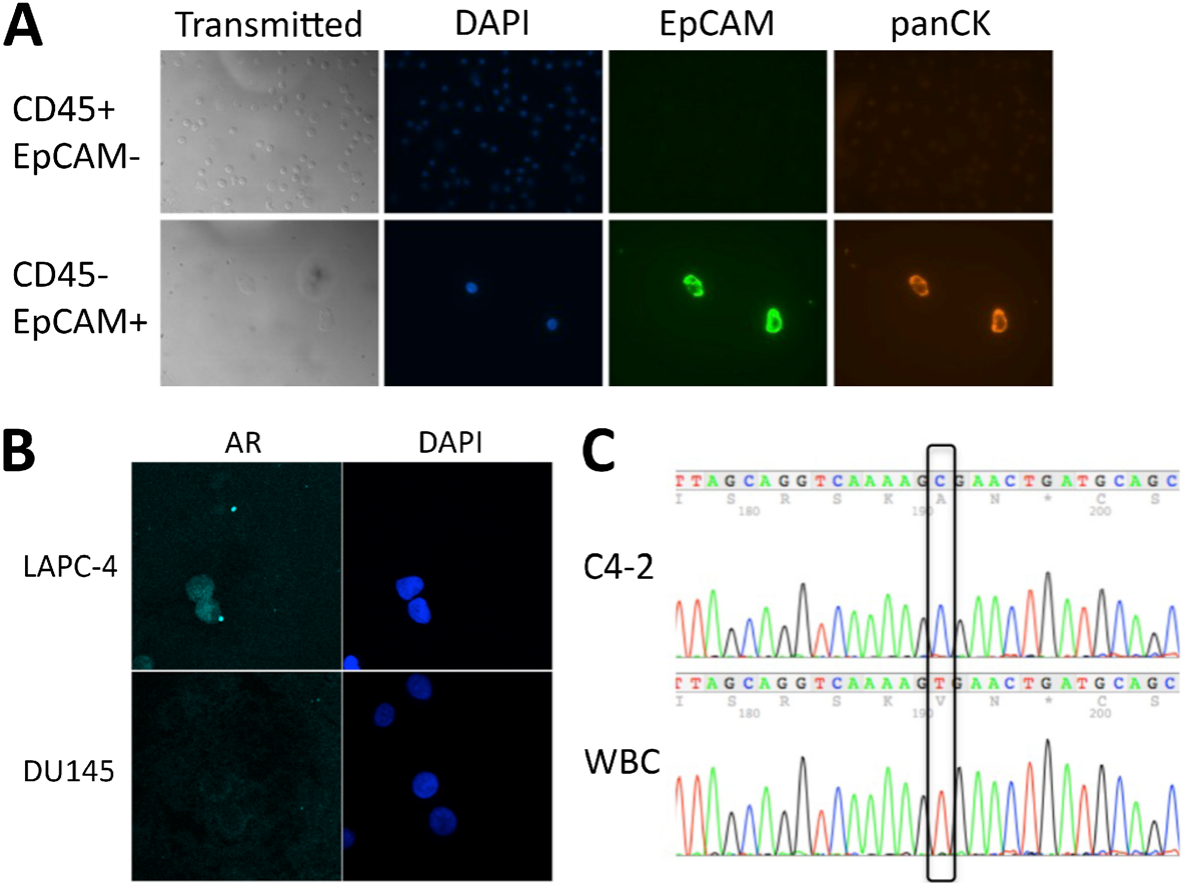
**Isolation and evaluation of cultured prostate cancer cells. A**. LAPC-4 prostate cancer cells spiked into whole blood from healthy donors were sorted onto slide chambers for immunofluorescence (IF) using flow-cytometry techniques based on expression pattern of CD45 and EpCAM. They were evaluated for presence of a nucleus with DAPI and expression of EpCAM and cytokeratin. **B**. AR-positive LAPC-4 cells and AR-negative DU145 cells spiked into whole blood from healthy donors were sorted onto slide chambers for IF and evaluated for AR staining and localization. **C**. Ten C4-2 cells spiked into whole blood from a healthy donor and 10 white blood cells (WBCs) were isolated by flow sorting, and extracted DNA underwent whole genome amplification. A portion of the AR gene was subsequently amplified and sequenced using capillary sequencing, and a known T→C mutation was identified.

The initial step in determining feasibility of androgen receptor (AR) characterization in CTCs involved spike-in experiments in which cells from the well-established prostate cancer cell line LAPC-4 were introduced into whole blood from healthy donors. After immunostaining for epithelial (EpCAM) and white blood cell (WBC) markers (CD45), EpCAM+/CD45- events were isolated using FACS and sorted onto slide chambers for immunofluorescence (IF) (Figure 1A). Enucleated cell debris was among the events sorted by FACS, but IF confirmed the identity of DAPI+/EpCAM+/CD45- cancer cells (Figure 1A). These cells were also found to express pan-cytokeratin, further supporting their identity as prostate cancer cells (Figure 1A). Of note, with these methods, using prostate cancer cells spiked into volunteer blood, there was large loss of cancer cells throughout the process (up to 90%, data not shown). As the focus of our study was on molecular characterization, not enumeration, we proceeded with these methods despite large loss and potential lack of enumeration sensitivity. To determine the feasibility for these methodologies in interrogating AR protein expression, similar spike-in experiments were performed with the LAPC-4 human prostate cancer cell line, which is known to overexpress AR [28]. As expected, AR was visualized and found to be nuclear in the AR-positive cell line LAPC-4, but absent in the AR-negative prostate cancer cell line DU145 (Figure 1B).

To further confirm the specificity of FACS-sorted events, C4-2 cells harboring a known mutation in AR were spiked into whole blood from healthy donors. As few as ten EpCAM+/CD45- C4-2 cells were sorted into PBS, and DNA was amplified using multiple displacement amplification. A portion of the *AR* gene was amplified using targeted PCR from the amplified whole genome DNA of sorted C4-2 cells and WBCs. Capillary sequencing confirmed the known A→G point mutation in C4-2 events with no detectable contamination from WBCs (Figure 1C). Thus, with these FACS based methodologies, CTC isolation and interrogation with specificity is feasible.

### Circulating tumor cells isolation from patients with mCRPC using FACS

**Figure 2:**
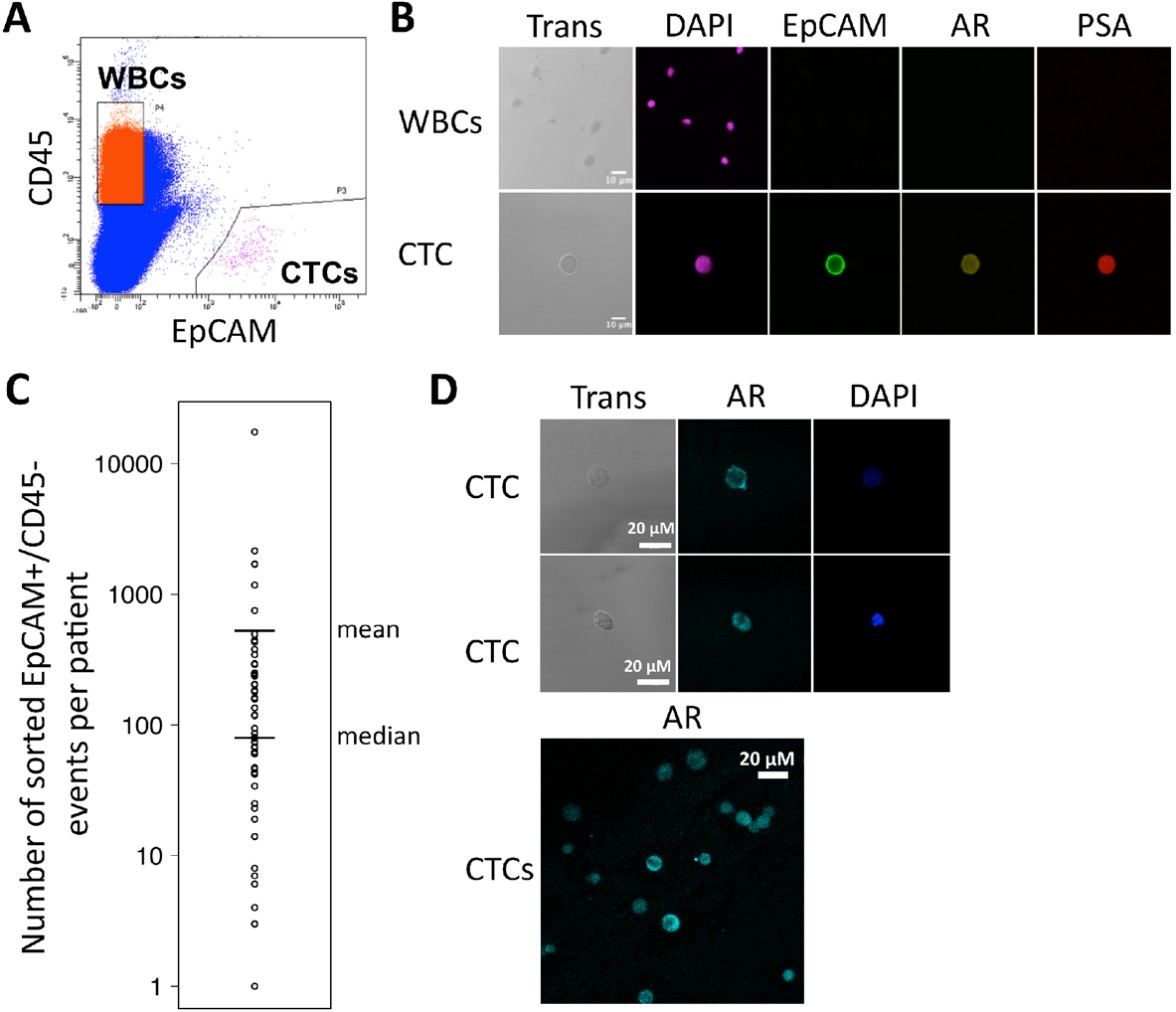
**Isolation of circulating tumor cells from patients with metastatic castration-resistant prostate cancer. A**. Plot of gating strategy applied on a representative patient sample capturing CD45+/ Epithelial Cell Adhesion Molecule (EpCAM)- WBCs and EpCAM+/CD45- CTCs. **B**. CTCs and WBCs were sorted onto slide chambers for IF using FACS-based techniques. Cells were stained for DAPI, EpCAM, AR, and Prostate Specific Antigen (PSA). **C**. The number of events for each of 51 patients who had EpCAM+/CD45- events sorted by FACS-based techniques. The mean (659) and median (80) number of events are indicated. **D**. Representative CTCs evaluated by IF demonstrating AR staining. The lower panel shows variable AR staining patterns.

To determine the applicability of these FACS-based isolation techniques for interrogation of CTCs from patients with mCRPC, 15 mL of whole blood was collected and EpCAM+/CD45- CTCs were isolated from an initial feasibility cohort of 15 patients (Table 1, Figure 2A). All patients had documented castration-resistance, and all were progressing on their current therapy. EpCAM+/CD45- events were isolated from 13 of 15 (87%) patients, and five or more events were isolated from 11 of 15 (73%) subjects. For those with isolated events, the range was 3–1700, with a mean of 335 and a median of 237 events.

**Table 1:** **Clinical characteristics of the feasibility cohort**

**Unique ID**	**Age (years)**	**PSA (ng/mL)**	Prior therapies*	**Events on FACS**	**Confirmed CTCs by IF**
11001	61	1077	L, B, K, O, D, M	1700	22
11002	80	787	L, D+/−Da	294	17
11003	88	124.3	L, B	94	0
11004	76	201.3	L, T, B, N, D	0	0
11005	80	741	L, F, D, M, E vs pl	4	1
11006	58	168.1	L, B, D	0	0
11007	62	34.24	L, B, D, E	3	0
11008	63	1971	L, B, D, M + I	751	17
11009	62	68.45	L, B, D, M	60	4
11010	79	2229	L, B, D, M, Ctx	62	0
11011	71	218.9	L, B, D+/− Af, E	345	23
11012	70	1425	L	290	25
11013	72	50.15	L, B, F, K, O	431	22
11014	81	25.5	L, B, O, T	237	10
11015	80	70.8	L, B, Di, D, S	86	3

*Prior therapies: A = aflibercept, B = bicalutamide, C = cyclophosphamide, Ci = cixutumumab, D = docetaxel, Da = dasatinib, Di = diethylstilbestrol, E = enzalutamide, F = flutamide, K = ketoconazole, L = LHRH agonist, M = mitoxantrone, N = nilutamide, O = orteronel, P = placebo, S = samarium lexidronam, T = tasquinimod.

To confirm the identity of FACS-sorted events as CTCs, EpCAM+/CD45- events were sorted directly onto a chamber slide for multiplex immunofluorescence (IF) staining for EpCAM, prostate specific antigen (PSA) and AR followed by visualization with fluorescence microscopy (Figure 2B). A CTC was defined as a visualized cell that was EpCAM+/CD45- by IF, and with an intact nucleus (DAPI positive). Of the 13 patients with events sorted by FACS, 10 had CTCs visualized on slide chambers according to this definition. We were unable to visualize CTCs from any patients with fewer than 60 sorted EpCAM+/CD45- events. Furthermore, FACS followed by IF staining and manual visualization lead to an approximate 90% loss of CTC events; roughly 10% of events identified by FACS were visualized on the chamber slide (Table 1). Despite loss through the FACS-based processes, CTC isolation from patients with progressive mCRPC was determined to be feasible as CTCs were isolated in the majority of patients. We next sought to utilize these methods for CTC isolation and interrogation in an additional cohort of patients. Peripheral blood was drawn for isolation of CTCs from 42 patients (Additional file 2: Table S1). Clots formed for one sample, preventing isolation of CTCs. Of the remaining 41 patients, CTC events were sorted from 38 patients. In both cohorts taken together, CTC events were identified and isolated in 51 of 56 (91%) evaluable patients utilizing FACS-based methodologies (median number of events 80, mean number of events 659; Figure 2C).

### Analysis of AR protein expression and localization

AR continues to play a central role in late-stage prostate cancer, even after the development of castration-resistance, as demonstrated by the recent FDA approval of AR targeted therapies to treat progressive mCRPC. Our initial effort was to therefore test the feasibility of our FACS-based sorting methods for interrogation of the *AR*. We were able to visualize AR expression, and 100% (10/10) of patients had CTCs with greater fluorescence intensity (expression) in comparison to the AR-negative DU145 cell line (Figure 1B). Fifty percent had AR staining intensity higher than AR-positive LAPC-4 cells (representative patient samples, Figure 2D). Qualitatively, the majority of AR staining within CTCs was intranuclear in subcellular localization; however, there appeared to be variability in AR staining intensity (expression) within and between patients (Figure 2D).

**Figure 3:**
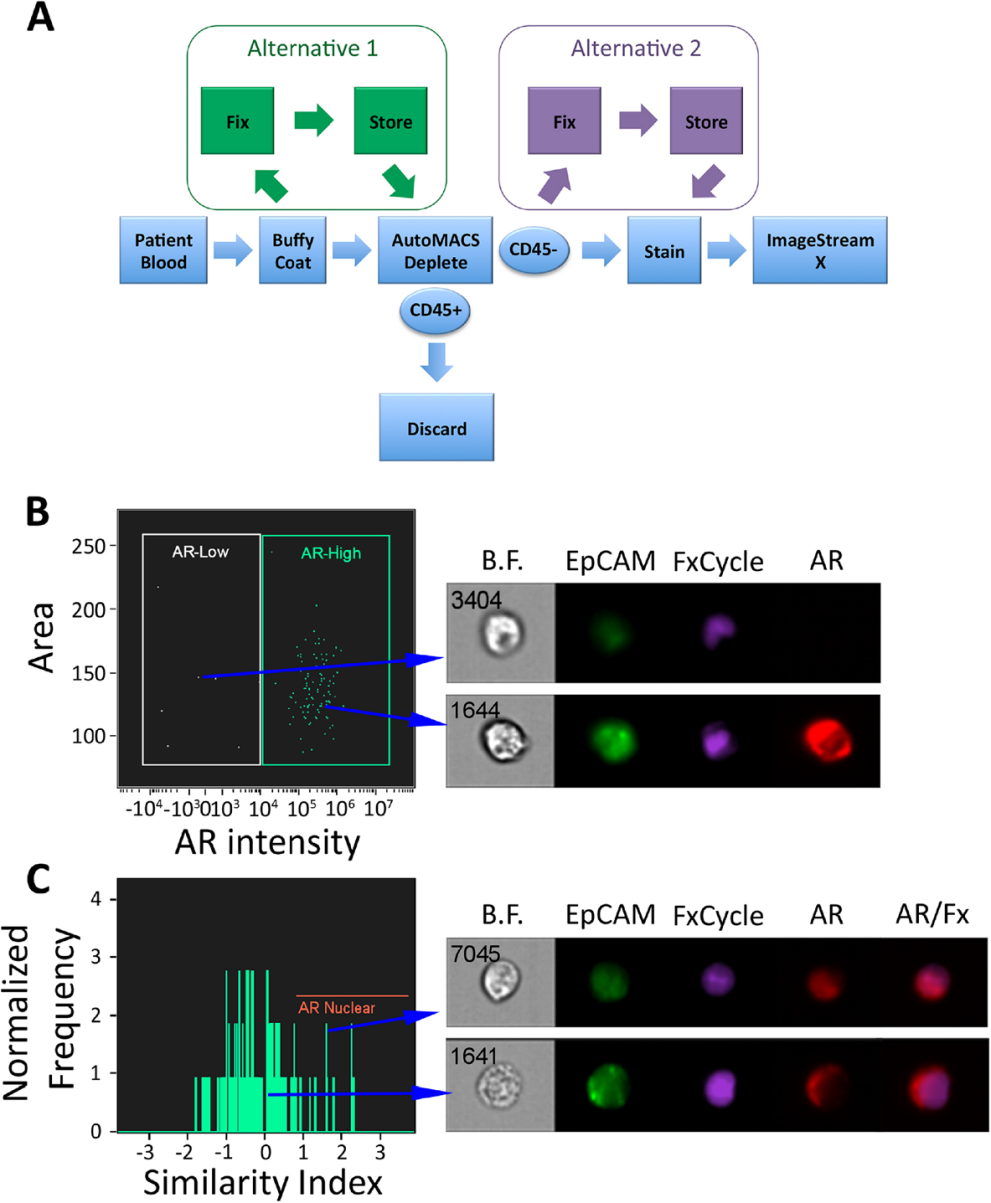
**AR protein interrogation in CTCs from patients with metastatic castration-resistant prostate cancer using ImageStreamX. A**. Flow chart illustrating the protocol for preparing CTCs for analysis using ImageStreamX. **B**. Representative dot plot showing area (cell size) and AR intensity (expression) for a single patient. Two CTCs have been selected to show corresponding images acquired using ImageStreamX technology, which demonstrate AR-Low (top panel) or AR-high (bottom panel) expression. **C**. Representative histogram showing nuclear or non-nuclear AR for all CTCs from a single patient. Nuclear AR is calculated by the similarity of AR staining and FxCycle Violet staining, and defined as Similarity Index >1. Two CTCs have been selected to show corresponding images acquired using ImageStreamX technology.

Although FACS followed by IF microscopy is feasible and can demonstrate qualitative differences in protein expression, these methods had several limitations, including loss of cells when sorting onto a slide chamber, labor-intensive protocols, and inability to obtain robust quantitative data. We therefore shifted to utilizing the ImageStreamX flow cytometry system for a more comprehensive and quantitative analysis of AR protein expression and localization. ImageStreamX combines flow cytometry with microscopy to produce multiple high resolution images of each cell in real-time [25-27]. Fixing and storing samples is also an option, which offers flexibility in staining and analysis (Figure 3A, Additional file 3: Figure S2). Finally, in a small pilot study of spike-in cancer cells in another malignancy, ImageStreamX had similar cell enumeration performance in comparison to the FDA approved CELLSEARCH system [25].

Within whole blood, CTCs are typically outnumbered by WBCs by several logs. To reduce noise from the vastly more abundant WBCs, and to improve visualization of CTCs, CD45+ cells were depleted using AutoMACS (Figure 3A). Evaluation of depleted cells confirmed that the vast majority of events are CD45+/EpCAM- as expected. There are however rare CD45+/EpCAM+ events within the depleted fraction; visualization of these double-positive events suggested that these events were the result of non-specific EpCAM staining of debris in association with CD45+ cells and thus were false positive events (Additional file 4: Figure S3A). To eliminate these events we utilized the Delta Centroid XY feature of ImageStreamX. This feature compares the proximity of two stains, in this case EpCAM and FxCycle Violet nuclear stain, to separate viable cells from debris. Rare cell cluster events were noted; however these were clusters of EpCAM + CTCs along with EpCAM- white blood cells. The intimate nature of these cancer and non-cancer clusters confounded our molecular characterization of these cell clusters. We thus also used size criteria to exclude large clusters of cells and debris (Additional file 4: Figure S3B).

We define true cells as Delta Centroid XY-gated, area-gated events that are nucleated with an intact membrane (Additional file 4: Figure S3C). Visualization of ImageStreamX-captured images confirmed that 94.2% of these events are indeed intact legitimate cells, the remainder being comprised of likely cell debris (Table 2). Furthermore, we define an ImageStreamX event as a CTC if it is a true cell that passed CD45 depletion and is EpCAM+. With these methods, 20 of 20 patients had at least one CTC on 29 of 31 blood draws. For all blood draws, there was a median of 23 (0–411) and mean of 51 (s.d. 82) CTCs (Table 2). Importantly, EpCAM+/CD45-depleted cells were larger than EpCAM-/CD45-depleted cells (Additional file 5: Figure S4), supporting their identity as CTCs [13,29-31].

**Table 2:** **Clinical characteristics and cell-staining characteristics of patients whose CTCs were evaluated by ImageStreamX**

**Patient ID**	Prior therapies*	**PSA (ng/mL)**	**EpCAM + Events**	**CTCs (%)**	**Mean AR intensity** ^**#**^ **(STDev)**	**Median AR intensity** ^**#**^ **(Range)**	**AR-high CTCs (%)**	**Mean SI (STDev)**	**Median SI (Range)**
12077	L, B	168.4	5	4 (80)	117262 (120651)	89033 (29879–322996)	5 (100)	0.67 (1.67)	−0.09 (−0.43-3.55)
12076	L, B, K, A	41.16	115	114 (99)	337937 (286897)	253104 (−6509-1427349)	108 (94)	−0.12 (0.85)	−0.25 (−1.79-2.31)
12079	L, B, S	32.24	104	96 (92)	25456 (36815)	11486 (1096–193627)	63 (61)	0.72 (1.06)	0.57 (−0.78-4.27)
12078	L, B, K	11.12	33	27 (82)	3202 (12736)	216 (−9849-56081)	5 (15)	0.97 (1.21)	0.95 (−0.98-2.06)
12080	L, B, D, C, A, E	554	0	NA	NA	NA	NA	NA	NA
12081	L, D, C	275.4	36	24 (67)	33078 (58419)	4115 (−12080-240098)	16 (44)	−0.05 (1.51)	−0.44 (−1.77-3.79)
12082	B, S	5.78	18	11 (61)	59487 (213913)	1437 (−6856-913784)	6 (33)	0.39 (0.94)	0.21 (−0.62-1.64)
12083	L, B, A, E	522.3	12	11 (92)	38260 (29857)	30748 (3725–92097)	9 (75)	0.3 (1.11)	−0.21 (−0.98-2.39)
12084	L	164.1	24	16 (67)	78782 (214426)	795 (−5122-800180)	8 (33)	0.6 (1.6)	0.17 (−1.02-3.63)
12081	L, D, C	382.6	15	5 (33)	−2323 (4049)	−2592 (−10466-4384)	0	NA	NA
12079	L, B, S	39.3	0	NA	NA	NA	NA	NA	NA
12086	B, L, K, D, A, E, Sa	1152	121	116 (96)	5674 (17971)	4256 (−13238-182505)	13 (11)	0.39 (0.66)	0.6 (−0.93-1.13)
12087	L, B	20.35	35	35 (100)	−11351 (21968)	−7673 (−68443-33422)	4 (11)	−0.46 (0.63)	−0.31 (−1.33-0.12)
12086	B, L, K, D, A, E, Sa	1320	11	11 (100)	115581 (88954)	102918 (46540–360131)	11 (100)	−0.36 (0.44)	−0.45 (−0.9-0.64)
12088	L, B, D	0.06	21	18 (86)	254074 (980505)	3184 (−1368-4502881)	4 (19)	−0.2 (2.65)	0.99 (−4.17-1.38)
12028-101	L, B, D, A, E	37.23	50	48 (96)	241547 (1561372)	4910 (−3059-11054469)	12 (24)	0.58 (1.51)	0.35 (−1.88-3.73)
12064-102	L, B, K, A, C	6.33	4	3 (75)	55055 (63046)	44257 (1328–130379)	2 (50)	3.74 (0.35)	3.74 (3.49-3.99)
12072-103	B, L, A, D	20.59	220	215 (98)	21851 (48606)	7965 (−45736-465037)	92 (42)	−0.1 (0.64)	−0.17 (−1.2-2.06)
12080-104	L, B, D, C, A, E	1040	3	2 (67)	81694 (72851)	108774 (−819-137127)	2 (67)	2.36 (0.43)	2.36 (2.06-2.67)
14093	Ci/Et, To, Et/Te	1.83	7	6 (86)	5566 (12133)	2578 (−8042-29243)	2 (29)	−0.12 (0.36)	−0.12 (−0.38-0.13)
12027-105	L, B, N, K, D/Da, A, E, R	489.8	40	40 (100)	7995 (31246)	3127 (−62338-124132)	5 (13)	1.32 (0.87)	1.65 (0.3-2.34)
14093	Ci/Et, To, Et/Te	1.98	23	22 (96)	500281 (2345906)	3774 (−9141-11261273)	8 (35)	−0.33 (1.54)	0.06 (−3.94-1.17)
12064-102	L, B, K, A, C	6.84	411	408 (99)	11281 (21967)	7020 (−6031-267789)	115 (28)	0.74 (0.52)	0.8 (−0.75-1.82)
12072-103	B, L, A, D	25.8	30	30 (100)	12437 (37826)	4871 (−19268-203291)	8 (27)	0.34 (0.68)	0.19 (−0.29-1.78)
12088	L, B, D	0.06	100	99 (99)	5505 (15912)	1670 (−13453-118622)	13 (13)	0.01 (0.61)	−0.17 (−0.89-1.3)
14092	L, B, K, N, A, R	498.7	13	11 (85)	51770 (97952)	689 (−60783-230484)	5 (38)	1.86 (0.82)	2.03 (0.48-2.6)
12028-101	L, B, D, A, E	46.31	29	27 (93)	23411 (69628)	4050 (−5317-359215)	7 (24)	0.92 (1.22)	0.37 (−0.62-2.85)
12076-106	L, B, K, A, D	9.33	59	59 (100)	29569 (74779)	9686 (−24144-356079)	29 (49)	1.09 (0.65)	1.05 (−0.11-3.43)
14090	L, B, N, S, A	6.81	10	8 (80)	41168 (90507)	−4692 (−83596-201844)	4 (40)	2.38 (0.52)	2.38 (1.76-3.02)
14089	L, B	5.06	11	9 (82)	192342 (687361)	−2246 (−149355-2254943)	3 (27)	2.33 (1.29)	1.71 (1.47-3.82)
14089	L, B	5.87	8	2 (25)	1953000 (5025861)	218121 (−25632-14383254)	6 (75)	1.02 (1.71)	1.53 (−2.35-2.49)

Thirty-one blood draws from 20 patients were evaluated by ImageStreamX technology, of which 29 blood draws from 20 patients had CTCs identified. SI = similarity index.

*Prior therapies: A = abiraterone, B = bicalutamide, C = cabazitaxel, Ci = cisplatin, D = docetaxel, Da = dasatinib, E = enzalutamide, Et = etoposide, K = ketoconazole, L = LHRH agonist, N = nilutamide, R = radium223, S = sipuleucel-T, Sa = samarium, Te = temozolemide, To = topotecan.

^#^Negative values are due to compensation within ImageStreamX.

Although enumeration of CTCs is possible with ImageStreamX, the rationale for use of this platform in this study was to characterize CTCs at the molecular level. Specifically, ImageStreamX functionality permitted further interrogation of AR within CTCs. Importantly, we used the LAPC-4 prostate cancer cell line as a control to define AR staining for these methods, since these cells express the wild-type AR [32] (Additional file 6: Figure S5). When LAPC-4 cells are treated with AR-agonist R1881, AR protein is stabilized leading to increased expression; whereas with AR-antagonist enzalutamide, AR expression decreases [33]. Based on these treatments, we defined “AR-high” within CTCs as AR intensity greater than 1 × 10^4^ (Additional file 6: Figure S5).

Upon binding to androgen ligand, activated AR is released from heat shock proteins and translocates to the nucleus to activate gene transcription [34]. Therefore, nuclear localization of AR can serve as a marker suggestive of AR activation. To quantitatively evaluate localization of AR in CTCs using ImageStreamX, we compared the overlaying pixel-by-pixel similarity of AR staining within the “AR-high” cells with FxCycle Violet nuclear staining, defined as the Similarity Index. As anticipated, LAPC-4 cells with the AR-agonist R1881 causes stability and nuclear localization of the AR, whereas the AR-antagonist enzalutamide leads to decreased AR protein levels [33]. LAPC-4 cells exposed to R1881 demonstrated a similarity index of 1 or greater, confirming nuclear localization (Additional file 6: Figure S5), and this cutoff was therefore used to demonstrate AR nuclear localization in patient samples. Direct visualization of CTCs from CRPC patients with AR staining validated this AR expression cut-off and nuclear localization threshold in patient samples (Figure 3B,C). We tested the reliability and reproducibility of these AR metrics by examining intra-patient variability among biological replicates of human CTCs using ImageStreamX. We imaged CTCs in three subjects, each of whom underwent two blood draws within six weeks and had at least five CTCs. Although the total number of CTCs varied between draws, the median AR expression and percentage of CTCs with high AR staining did not vary widely between blood draws (Additional file 7: Table S2).

**Figure 4:**
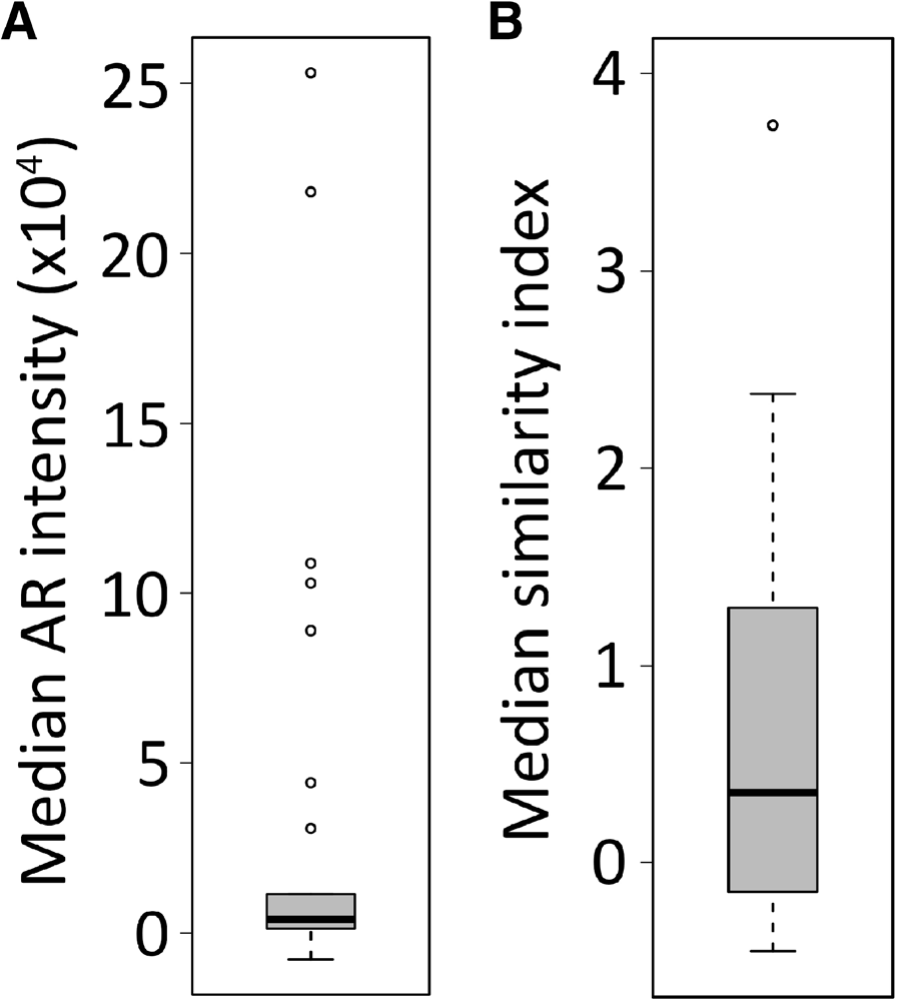
**Heterogeneous AR expression intensity and localization in patients with advanced prostate cancer.** Plots showing the **(A)** median AR intensity or **(B)** median similarity index of CTCs from 29 blood draws from 20 patients with advanced prostate cancer.

Using ImageStreamX, within a cohort of 20 patients, AR staining of patient CTC samples demonstrated inter-subject variability in expression and localization of AR (Table 2, Figure 4A,B). For the entire population the median AR intensity was (38260) with a very wide range of (−11,351 - 1,953,000). Furthermore, as exemplified in Figure 3B, within a patient the AR expression was variable; populations of CTCs expressing high levels of AR were visualized within the same sample with low AR expressing CTCs. Nuclear localization of AR showed similar population differences within a patient (example Figure 3C). This suggests that a patient may have heterogeneous CRPC biology with respect to AR.

### Association of prior therapy and proliferation status with AR characterization

**Figure 5:**
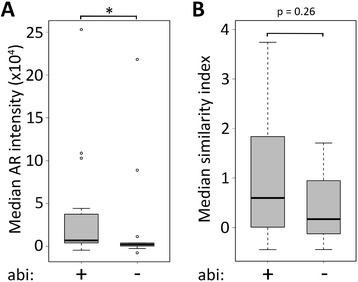
**Association between prior exposure to abiraterone and increased AR expression.** Box-and-whisker plots showing **(A)** median AR intensity or **(B)** median similarity index of CTCs from patients with prior exposure to abiraterone versus no prior exposure to abiraterone (abi = abiraterone) (* p-value <0.05).

To further test whether AR expression and nuclear localization may be clinically relevant, we sought to examine AR biology as it relates to prior CRPC therapy. This was a clinically diverse cohort of mCRPC patients (Table 2), with variable exposure to potent AR targeted therapies, including the androgen synthesis inhibitor abiraterone acetate. Within our cohort of 20 patients, 10 had previously received abiraterone, and 6 had received the AR antagonist enzalutamide, all 6 of whom had also received abiraterone. We sought to test the hypothesis that patients whose disease had progressed despite abiraterone would have altered AR expression within CTCs. For example, we would hypothesize that after failure of this AR targeted therapy, CTCs may have lower AR expression and less nuclear (active) AR if a non-AR mediated pathway of clinical resistance is evident. Interestingly, there was a significant increase in AR expression within CTCs from patients with prior abiraterone treatment and progression (N = 10) compared to abiraterone naïve patients (N = 10) (median AR staining intensity 7020 [Interquartile range 4153–37503] vs. 2124 [216–4115], p < 0.05). There was no significant change in cellular localization pattern within CTCs from patients with prior abiraterone exposure (Figure 5). These data suggest compensation by cancer cells after exposure to abiraterone by increasing AR expression. There were no patients who received enzalutamide but not abiraterone, and AR expression level in those who progressed on both abiraterone and enzalutamide was similar to that of patients who progressed on abiraterone but were naïve to enzalutamide (median 4910 [IQR 4153–66833] *vs* 7020 [4153–37503], p = 0.97).

**Figure 6:**
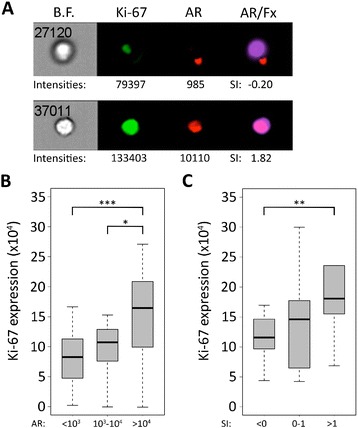
**Correlation of AR expression or nuclear localization with expression of the proliferation marker Ki-67. A**. Representative images of two cells acquired with ImageStreamX that were multiplex stained for Ki-67, AR, and FxCycle Violet. Images represent cells with low AR expression, non-nuclear AR, and low Ki-67 staining (top panel) or high AR expression, nuclear AR, and high Ki-67 staining (bottom panel). **B**. Box-and-whisker plot showing the Ki-67 expression of AR-low (AR intensity <10^3^), AR-intermediate (AR intensity 10^3^ – 10^4^), and AR-high (AR intensity >10^4^) CTCs from all samples that had Ki-67 staining and cells in all three AR expression groups (n = 16 patients). **C**. Box-and-whisker plot showing the Ki-67 expression of CTCs with low similarity index (SI <0), intermediate similarity index (SI 0–1), or high similarity index (SI >1) from all samples that had Ki-67 staining and cells in all three similarity index groups (n = 10 patients) (* p-value <0.05, ** p-value <0.01, *** p-value <0.001).

To determine if AR remains a driver of proliferation in this heterogeneous cohort of patients with mCRPC, we also examined co-expression of AR and Ki-67, a well-established marker of proliferation (Additional file 8: Figure S6A-C) [35]. Interestingly, AR-high cells expressed higher levels of Ki-67 than AR-low cells (Figure 6A,B). Similarly, AR positive cells with nuclear AR localization expressed higher levels of Ki-67 than cells with cytoplasmic AR localization (Figure 6A,C). These data depict an association between AR expression and nuclear localization and cancer cell proliferation in patients with advanced prostate cancer, even in the setting of castration-resistance and progression despite multiple AR-targeted therapies.

## Discussion

A major unmet need in personalization of prostate cancer care is the identification and validation of therapeutic biomarkers. One major limitation in our ability to develop biomarkers for mCRPC therapies is the difficulty in obtaining patient tumor specimens that are reflective of their current disease biology. CTC interrogation offers a potential source of such biomaterial; however, evaluation of CTCs is logistically difficult, and to date, there are no established methods for the study of CTCs at the molecular level. The principal goal of this study was to develop a novel, logistically feasible and robust method for interrogating CTCs from patients with progressive CRPC. Given the central importance of AR biology in prostate cancer therapeutics, our focus was on utilizing these techniques to study AR within CTCs.

We demonstrate the feasibility of two different flow cytometry-based techniques for the evaluation of CTCs from blood of men with progressive mCRPC. Our approach is streamlined and uses equipment that is widely available. Furthermore, fixation of cells for ImageStreamX allowed for short-and-long term storage of cells, facilitating greater experimental flexibility, and templates used during sample acquisition and data analysis were able to be standardized. The specificity of the cells isolated and evaluated was confirmed subsequent to FACS CTC isolation. Direct visualization and staining of these events confirmed that these were cells that expressed EpCAM, PSA and AR, but not CD45, and contained a nucleus.

The focus of the majority of CTC evaluation to date has been enumeration. While this is informative and prognostic [36], CTC isolation with molecular profiling of advanced disease has the potential to advance the personalization of CRPC therapy. Clinical trials are beginning to tailor therapy to specific molecular subtypes, and the analysis of CTCs will be a vital tool in identifying which therapies are most appropriate for each patient.

To that end, our goal was to show feasibility of isolation and molecular profiling of CTCs from patients with mCRPC. Given the pivotal role of AR, we focused our analysis on the expression and subcellular localization of AR. We found that AR expression and localization is heterogeneous between patients, and even within a patient. The finding that patients have CTCs with pleiotropic AR expression and localization is novel and may suggest variable or incomplete efficacy of AR-targeted therapies for such patients. Whether specific clones are selected under the therapeutic pressure of specific agents is an obvious avenue of further investigation.

With our quantitative analysis of AR protein expression using the ImageStreamX platform, our results could have implications for future clinical research with embedded CTC biomarker interrogation. We initially hypothesized that after failure of abiraterone, patients would have less active AR (demonstrated by less AR expression or nuclear localization) supporting clinical progression through a non-AR directed mechanism. In fact, our data support that patients with prior exposure to abiraterone have higher AR protein expression and statistically unchanged AR nuclear localization compared to patients who were not exposed to abiraterone. This parallels previously reported xenograft models showing increased AR expression in prostate cancer after abiraterone [37,38]. Our data suggest that combining abiraterone with an AR antagonist such as enzalutamide may be effective in those patients, and especially in patients who still have nuclear, highly expressed AR. A large national phase III clinical trial of enzalutamide and abiraterone in combination is underway testing this hypothesis (NCT01949337). We also show, for the first time, that CTCs with nuclear AR have more Ki-67 staining compared to CTCs with cytoplasmic AR, implying that cells with nuclear AR are more proliferative. These results confirm that AR remains important in mCRPC patients despite clinical attempts to disrupt AR signaling. Thus, use of AR expression and localization in CTCs to guide management decisions warrants further investigation.

The parameters used to identify cells of interest are of concern when identifying rare events. We chose to focus on EpCAM + cells due to the wealth of experience supporting these selection criteria. It may well be, however, that there are additional markers which could allow identification/isolation of CTCs [39-41], which we are not collecting/isolating with our current techniques. One major benefit of the ImageStreamX multiplex protein interrogation approach is its flexibility; as our understanding of CTC biology becomes more refined, one could easily replace EpCAM-specific antibodies with other targeted markers. Of note, we did identify EpCAM-/AR + cells with ImageStreamX. These were noted to be smaller than the EpCAM + cells as a whole, leading to the presumption that the majority of these events are white blood cells that escaped negative selection. However, it is possible that they are EpCAM-low CTCs, and further evaluation of this population in particular is needed.

The relatively small sample size of this feasibility study is a limitation. A larger prospective study in a homogeneous patient population is needed to validate quantitative AR protein characterization within CTCs as a therapeutic biomarker. Fixed cells analyzed by ImageStreamX are not suitable for further interrogation of DNA or RNA expression. This is a limitation of our protein-based analysis platform. Of note, our initial FACS-based approach does allow for DNA interrogation with specificity, although this was not the focus of this current study.

There are many considerations when developing novel techniques for isolating and interrogating CTCs as potential biomarkers [13]. One primary question that remains largely unanswered is to what extent CTCs are representative of the metastatic disease. The fact that CTC enumeration is prognostic for CRPC suggests a link between CTC and metastatic disease burden, but to what extent the CTCs are in fact part of the metastatic process is not clear. One small study of prostate cancer patients showed a lymph node metastasis was phylogenetically more closely related to the CTCs and to one particular focus of the primary prostate tumor than other prostate tumor foci [42]. The field of CTC research is still in its relative infancy and new molecular techniques will enable further characterization of the relationship biologically between CTC and metastasis.

Other groups have performed highly elegant experiments examining CTC genomics and expression for other diseases [43,42]. In CRPC, reports of CTC molecular interrogation are limited, but have included proteins associated with epithelial to mesenchymal transition and genomic changes such as loss of PTEN or TMPRSS-ERG translocations [39,41]. IHC methods have also been described to ascertain AR expression in CTCs [44]. Furthermore, in a recent study, single-cell immunofluorescence was used to suggest whether CTCs had active or suppressed AR signaling, determined by PSA and PSMA, respectively, in untreated and CRPC patients [12]. Evaluation of AR isoform expression may have clinical value. A recent study showed that expression of the AR variant AR-v7 in CTCs may be associated with primary resistance to abiraterone and enzalutamide [45].

ImageStreamX analysis could easily be adapted to evaluate for expression of AR-v7 using anti-AR-v7 antibodies [8]. Of note, the AR antibody used in the present study is raised against a relatively N-terminal peptide, which is expected to bind to common truncated splice variants, including AR-V7. Mutation in the AR has also been demonstrated in mCRPC [46,47]. These mutation or ligand independent splice variations have been shown to lead to enhanced activation of the AR. Although our protein-centric methods do not allow for the detection of these specific genetic or mRNA events, ImageStreamX is able to quantify nuclear localization of the AR, which is a surrogate for active nuclear hormone receptor. Other groups have also published reports suggesting AR subcellular localization in CTCs has clinical value. In their study, Darshan et. al found that cytoplasmic AR status in CTCs correlated with favorable clinical response to taxane chemotherapy [48]. Our methods complement and expand on these studies utilizing quantitative methodologies to interrogate AR protein expression in CTCs.

In summary, our focus on AR protein analysis in CTCs has demonstrated that interrogation of AR expression and subcellular localization may have clinical relevance. Alterations in these metrics are associated with progression on abiraterone and increased expression of a marker of cellular proliferation. Given the continued focus on AR as a therapeutic target in mCRPC, quantitative analysis of AR expression and nuclear localization has the potential to serve as a predictive therapeutic biomarker. Further validation of our techniques, in the context of prospective clinical trials is warranted. As our ability to personalize prostate cancer management improves, streamlined CTC isolation and molecular profiling will become an integral piece in the management of advanced prostate cancer. Although we focus on AR expression for the purpose of this study involving patients with mCRPC, our quantitative multiplexing methods are adaptable for other pathways of interest within mCRPC and potentially broadly applicable to other metastatic malignancies.

## Conclusions

We provide molecular characterization of the AR signaling pathway in circulating prostate cancer cells. We have demonstrated feasibility of AR characterization, showing variation in AR expression and localization. AR expression and nuclear localization in CTCs may be a dynamic therapeutic biomarker worthy of future validation in a more homogeneous patient population in the context of an AR-targeted therapy. As methods for isolation and characterization of CTCs improve, CTCs may become established as a clinical tool to guide the management of advanced prostate cancer.

## Additional files

Additional file 1: Figure S1. Single stain compensation controls. Dot plots showing single stain compensation controls of VCAP or CWR22RV1 cells that were stained for EpCAM, AR, Ki-67, or FxCycle Violet. Additional file 2: Table S1. Numbers of CTCs isolated by FACS-sorting. Numbers of CTCs isolated for 42 patients, in addition to the feasibility cohort shown in Table 1. Additional file 3: Figure S2. Fixing cells does not alter EpCAM intensity. Flow cytometry dot plots showing control cells (left), or unfixed (middle) or fixed (right) LAPC-4 prostate cancer cells stained for EpCAM. Additional file 4: Figure S3. Use of ImageStreamX to confirm CTCs as EpCAM+, CD45-depleted cells. A. Patient cells were CD45-depleted using a biotinylated anti-CD45 antibody. CD45 positive cells were stained with Streptavidin-Alexa488 and cells were acquired on ImageStreamX. EpCAM and CD45 were mutually exclusive, and double positive events were cell debris or cell clusters. B. Gating using Delta Centroid XY feature eliminates debris. Representative images from a patient sample demonstrating that Delta Centroid XY high events are false positive and Delta Centroid XY low events are true CTCs. Events were gated on FxCycle + EpCAM + Delta Centroid XY+. C. Patient CTCs are EpCAM + FxCycle + and have an intact membrane. Representative images from two separate patients acquired through ImageStreamX showing events considered to represent viable CTCs (top panel) and those considered to represent cellular debris (bottom panel). Additional file 5: Figure S4. EpCAM + blood cells are larger than EpCAM- blood cells. Area of CD45-depleted, EpCAM + or EpCAM- cells. Area is calculated using ImageStreamX technology (*** p-value <0.001). Additional file 6: Figure S5. AR positive cells are defined by AR intensity of 10^4^ or greater. LAPC-4 prostate cancer cells were treated with the AR agonist R1881 (1nM, top panel) or the AR antagonist enzalutamide (10 μM, bottom panel). Cells were stained for intracellular AR and analyzed using ImageStreamX. LAPC-4 cells lose AR expression in the presence of the AR inhibitor enzalutamide, indicating the threshold for AR-high cells is AR intensity level 10^4^. Additional file 7: Table S2. CTCs from repeat blood draws analyzed by ImageStreamX. Table comparing intra-patient variability of the median AR intensity and similarity index for three patients whose blood was drawn at two different time points within six weeks. Additional file 8: Figure S6. Ki-67 intensity is higher in proliferating cells. Dot plots showing Ki-67 intensity of CWR22RV1 cells treated with (A) 1 μM or (B) 8 μM of mitomycin, which inhibits proliferation. C. Boxplot depicting Ki-67 intensity in each population (** p-value <0.01).

## Abbreviations

CTCs: Circulating tumor cells
mCRPC: Metastatic castration-resistant prostate cancer
FACS: Fluorescence-activated cell sorting
AR: Androgen Receptor
PCR: Polymerase chain reaction
