# Supplementary figures and images for: Quantitative characterization of androgen receptor protein expression and cellular localization in circulating tumor cells from patients with metastatic castration-resistant prostate cancer
Source: J Transl Med. 2014 Nov 26;12:313. doi: 10.1186/s12967-014-0313-z (PMC4252013; doi:10.1186/s12967-014-0313-z)

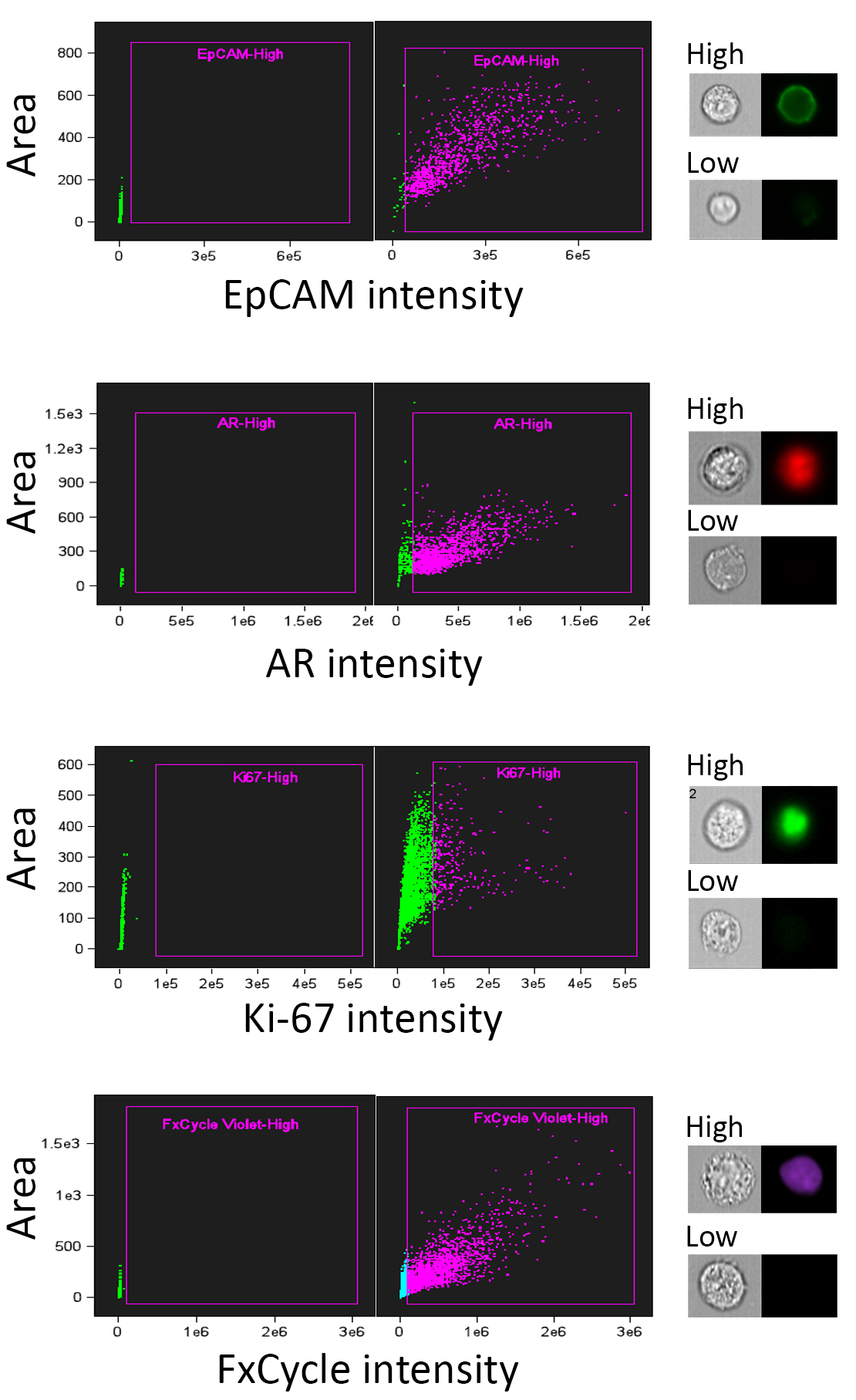

Supplement: Additional file 1: Figure S1. — Single stain compensation controls. Dot plots showing single stain compensation controls of VCAP or CWR22RV1 cells that were stained for EpCAM, AR, Ki-67, or FxCycle Violet. [file 12967_2014_313_MOESM1_ESM.jpeg]

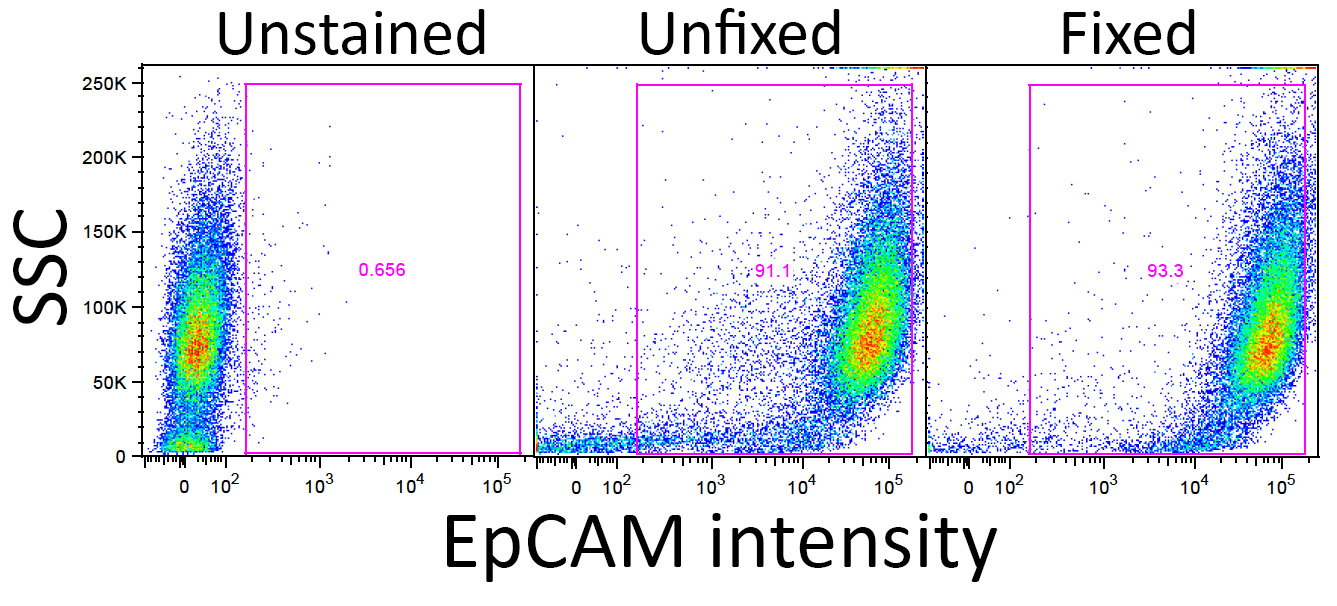

Supplement: Additional file 3: Figure S2. — Fixing cells does not alter EpCAM intensity. Flow cytometry dot plots showing control cells (left), or unfixed (middle) or fixed (right) LAPC-4 prostate cancer cells stained for EpCAM. [file 12967_2014_313_MOESM3_ESM.jpeg]

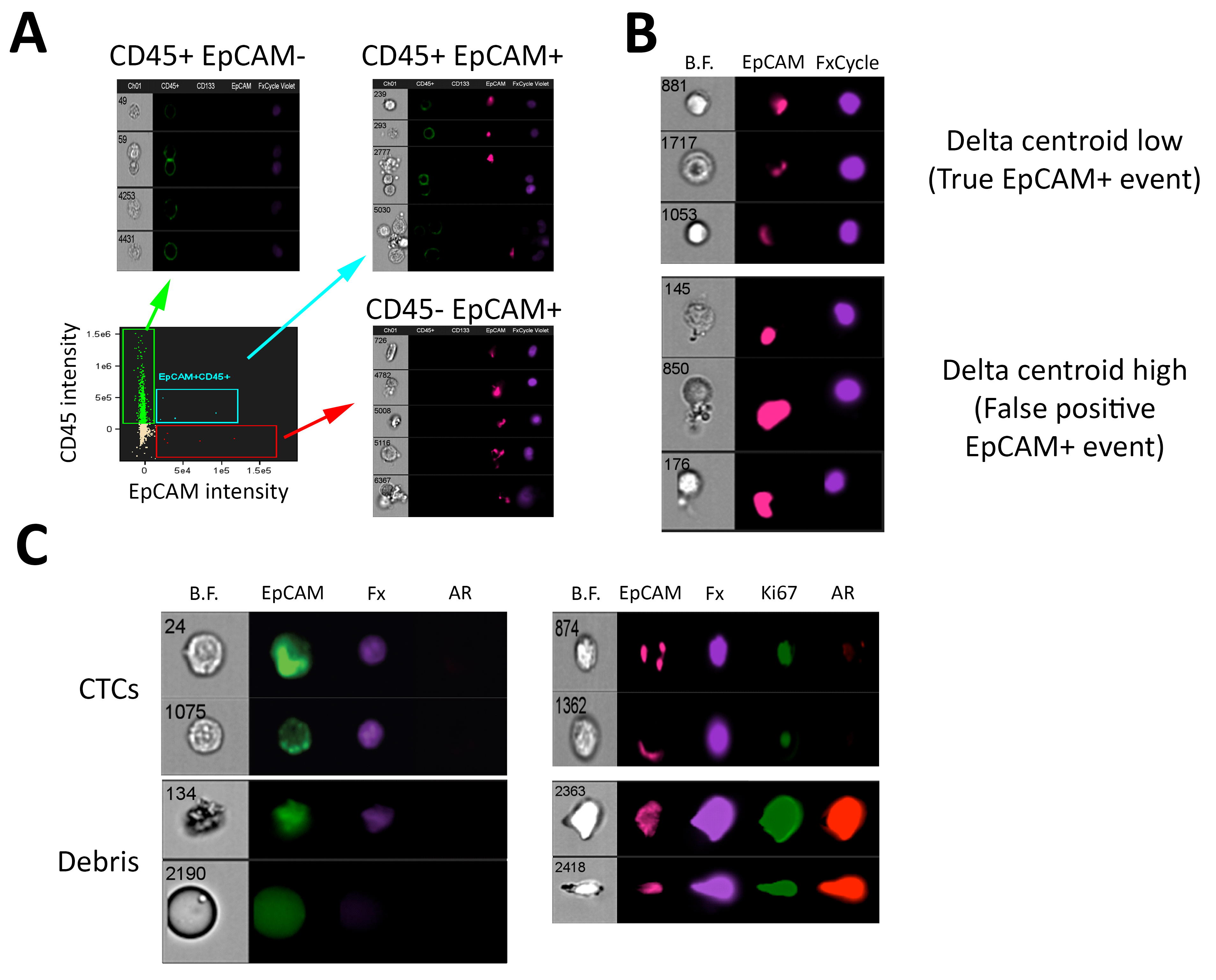

Supplement: Additional file 4: Figure S3. — Use of ImageStreamX to confirm CTCs as EpCAM+, CD45-depleted cells. A. Patient cells were CD45-depleted using a biotinylated anti-CD45 antibody. CD45 positive cells were stained with Streptavidin-Alexa488 and cells were acquired on ImageStreamX. EpCAM and CD45 were mutually exclusive, and double positive events were cell debris or cell clusters. B. Gating using Delta Centroid XY feature eliminates debris. Representative images from a patient sample demonstrating that Delta Centroid XY high events are false positive and Delta Centroid XY low events are true CTCs. Events were gated on FxCycle + EpCAM + Delta Centroid XY+. C. Patient CTCs are EpCAM + FxCycle + and have an intact membrane. Representative images from two separate patients acquired through ImageStreamX showing events considered to represent viable CTCs (top panel) and those considered to represent cellular debris (bottom panel). [file 12967_2014_313_MOESM4_ESM.jpeg]

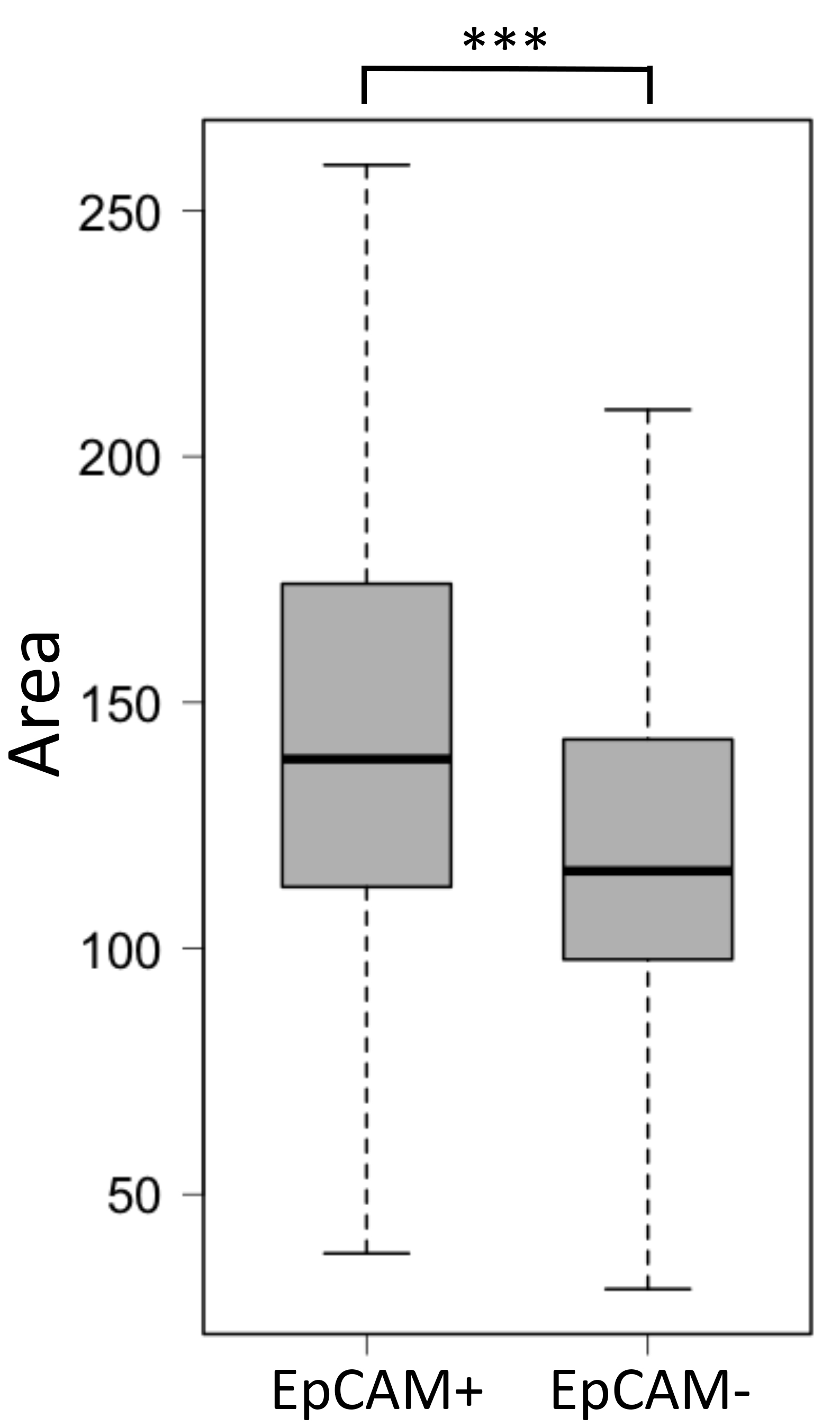

Supplement: Additional file 5: Figure S4. — EpCAM + blood cells are larger than EpCAM- blood cells. Area of CD45-depleted, EpCAM + or EpCAM- cells. Area is calculated using ImageStreamX technology (*** p-value <0.001). [file 12967_2014_313_MOESM5_ESM.jpeg]

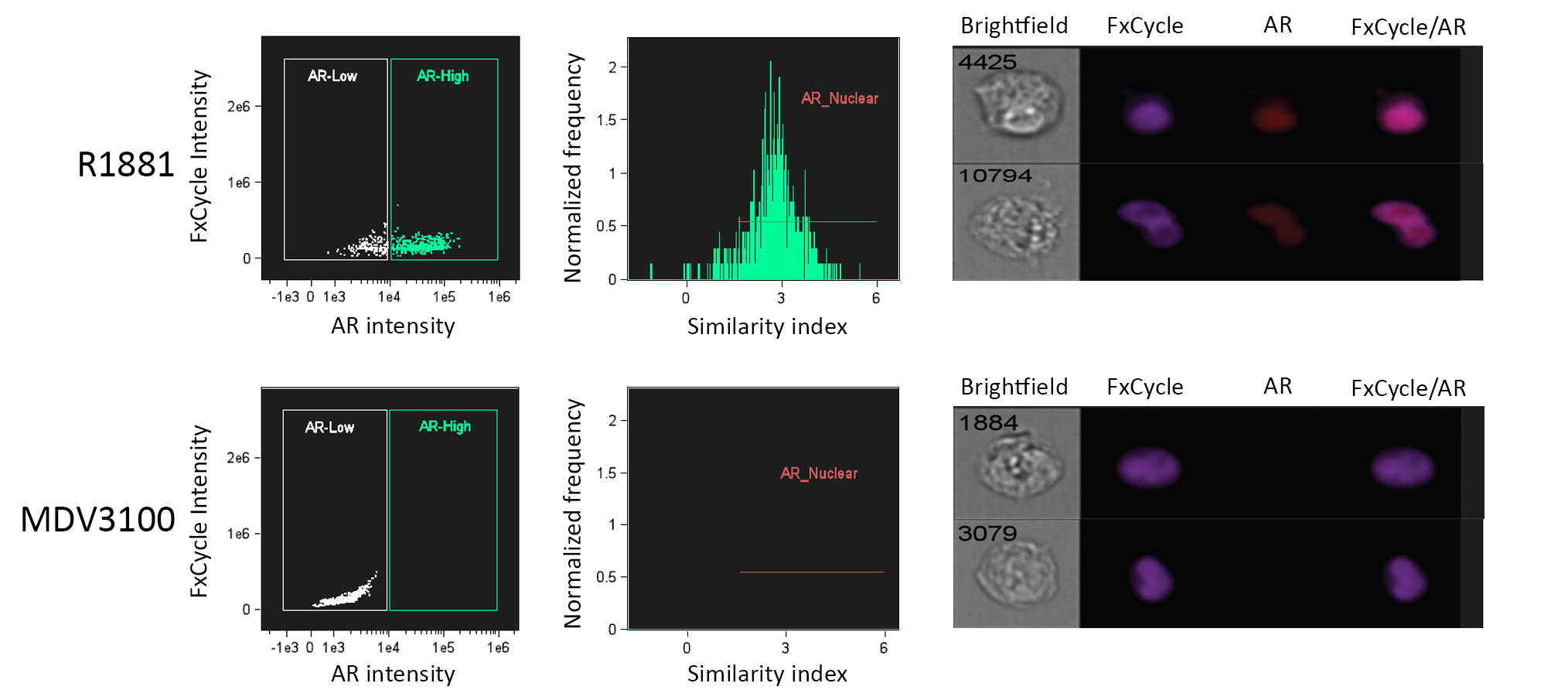

Supplement: Additional file 6: Figure S5. — AR positive cells are defined by AR intensity of 104 or greater. LAPC-4 prostate cancer cells were treated with the AR agonist R1881 (1nM, top panel) or the AR antagonist enzalutamide (10 μM, bottom panel). Cells were stained for intracellular AR and analyzed using ImageStreamX. LAPC-4 cells lose AR expression in the presence of the AR inhibitor enzalutamide, indicating the threshold for AR-high cells is AR intensity level 104. [file 12967_2014_313_MOESM6_ESM.jpeg]

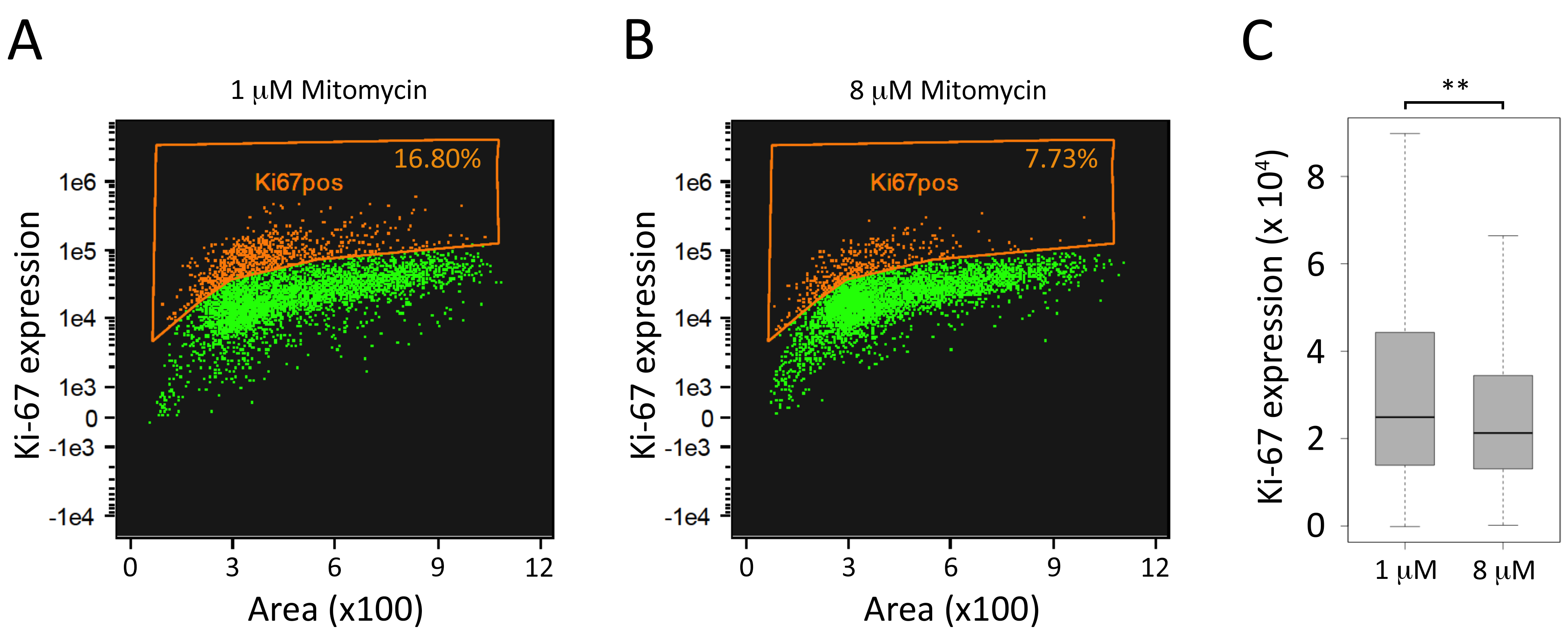

Supplement: Additional file 8: Figure S6. — Ki-67 intensity is higher in proliferating cells. Dot plots showing Ki-67 intensity of CWR22RV1 cells treated with (A) 1 μM or (B) 8 μM of mitomycin, which inhibits proliferation. C. Boxplot depicting Ki-67 intensity in each population (** p-value <0.01). [file 12967_2014_313_MOESM8_ESM.jpeg]
